# Supplementary material for: A Universal Spectrum Annotator for Complex Peptidoforms in Mass Spectrometry-Based Proteomics
Source: Anal Chem. 2025 Oct 14;97(42):23120–30. doi: 10.1021/acs.analchem.5c02832 (PMC12573231; doi:10.1021/acs.analchem.5c02832)
Supplement: Supplementary file 1 [file ac5c02832_si_001.pdf]

Supporting Information:

A universal spectrum annotator for complex peptidoforms in mass spectrometry-based proteomics

Authors:

Douwe Schulte, Rien W. Leuvenink, Shelley Jager, Albert J. R. Heck, Joost Snijder*

Affiliation:

Biomolecular Mass Spectrometry and Proteomics, Bijvoet Center for Biomolecular Research and Utrecht Institute of Pharmaceutical Sciences, Utrecht University, Padualaan 8, 3584CH Utrecht, The Netherlands

*Correspondence to: j.snijder@uu.nl

Supporting Information:

- Table S1: universal spectrum indices for all displayed spectra
- Figure S1: Annotation of multiple peptidoforms within chimeric scans
- Figure S2: Annotation statistics comparison
- Figure S3: Site-localization of post-translational modifications

Table S1: universal spectrum identifiers (USIs) for all used spectra with the used fragmentation mode. These can be used directly in the Annotator to view the spectra.

Figure	USI
2 EThcD	mzspec:PXD043489:20210331_F1_UM1_Peng0013_SA_139H2_Ingel_Thermolysin.raw:scan:3828:FGGGTKLELKR/3
3 EThcD	mzspec:PXD059727:search/EAD9_09.wiff2(1)/EAD9_09.wiff2(1).mgf:index:5327: GLTFQQN[GNO:G75079FY]ASSMC[Carbamidomethyl]VPDQDTAIR/4+GLTFQQN[GNO:G52512ZN]ASSMC[Carbamidomethyl]VPDQDTAIR/4
4 HCD	mzspec:PXD047679:20221122_EX3_UM7_Kadav001_SA_EXT00_16_DSS_11.raw:scan:40889:GGK[xlink:dss[138]#XLDSS]IEVQLK//KVESELIK[#XLDSS]PINPR/4
5 TD ETD	mzspec:PXD059727:26072023_MS2_TZB_1um_HiRes_Isol1284mzNarrow_ETD_RT5ms_6e6_IT200_deconvoluted.mzML:scan:1:EVQLVESGGGLVQPGGSLRLSC[C:Cystine#XL1]AASGFNIKDTYIHWVRQAPGKGLEWVARIYPTNGYTRYADSVKGRFTISADTSKNTAYLQMNSLRAEDTAVYYC[#XL1]SRWGGDGFYAMDYWGQGTLVTVSSASTKGPSVFPLAPSSKSTSGGTAALGC[C:Cystine#XL2]LVKDYFPEPVTVSWNSGALTSGVHTFPAVLQSSGLYSLSSVVTVPSSSLGTQTYIC[#XL2]NVNHKPSNTKVDKKVEPKSC[C:Cystine#XL3]DKT//DIQMTQSPSSLSASVGDRVTITC[C:Cystine#XL4]RASQDVNTAVAWYQQKPGKAPKLLIYSASFLYSGVPSRFSGSRSGTDFTLTISSLQPEDFATYYC[#XL4]QQHYTTPPTFGQGTKVEIKRTVAAPSVFIFPPSDEQLKSGTASVVC[C:Cystine#XL5]LLNNFYPREAKVQWKVDNALQSGNSQESVTEQDSKDSTYSLSSTLTLSKADYEKHKVYAC[#XL5]EVTHQGLSSPVTKSFNRGEC[#XL3]/1
S2 HCD	mzspec:MSV000082368:testing_plasma.spectrum.mgf:scan:24835:NEC[Carbamidomethyl]FIEHK/2+QYNVGPSVSK/2+EC[Carbamidomethyl]EVTHQGLSSPVTK/3
S3 EThcD	mzspec:PXD059727:search/Byonic_20241128_Z1_UM1_5858593_SA_EXT00_cIGA_EADonly_65ms_06.wiff2_Ogly/Byonic_20241128_Z1_UM1_5858593_SA_EXT00_cIGA_EADonly_65ms_06.wiff2_Ogly.mgf:scan:3835:HYTNPSQDVTVPC[Carbamidomethyl]PVPST[GNO:G14660IB]PPT[GNO:G14660IB]PS[GNO:G56558YT]PS[GNO:G14660IB]TPPTPSPSC[Carbamidomethyl]C[Carbamidomethyl]HPR/5

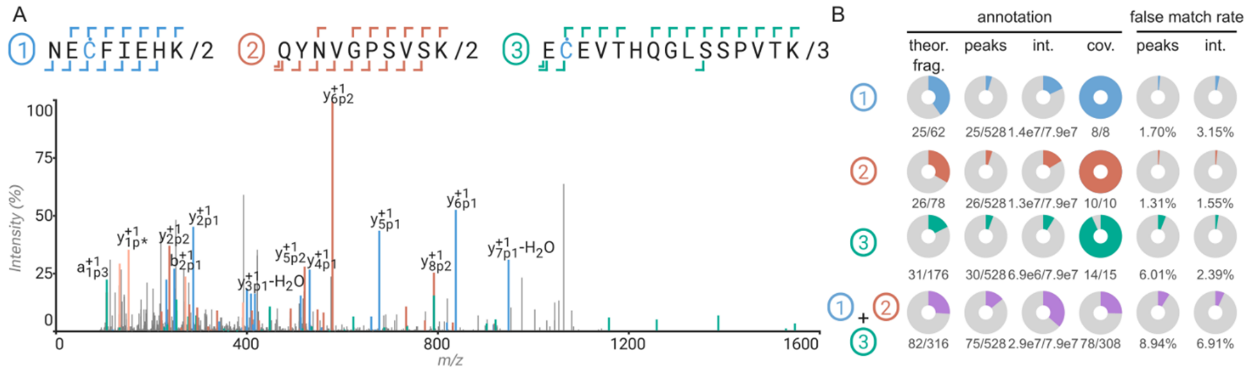

Figure S1: Simultaneous annotation of multiple peptidoforms within a DIA chimeric scan. A) Annotated chimeric HCD spectrum of three co-isolated peptidoform ions as identified by DeepNovo-DIA.[1] From tryptic digest of HeLa samples. B) Annotation statistics provided by Annotator for the individual peptidoforms and the combined set. Statistics include the fraction of theoretical fragments found, fraction of measured peaks annotated, fraction of MS2 intensity accounted for by annotated peaks, and fraction of sequence coverage. The false match rate (FMR) is estimated based on peak numbers and intensities following annotation with the same criteria after a random m/z shift is applied to the spectrum.

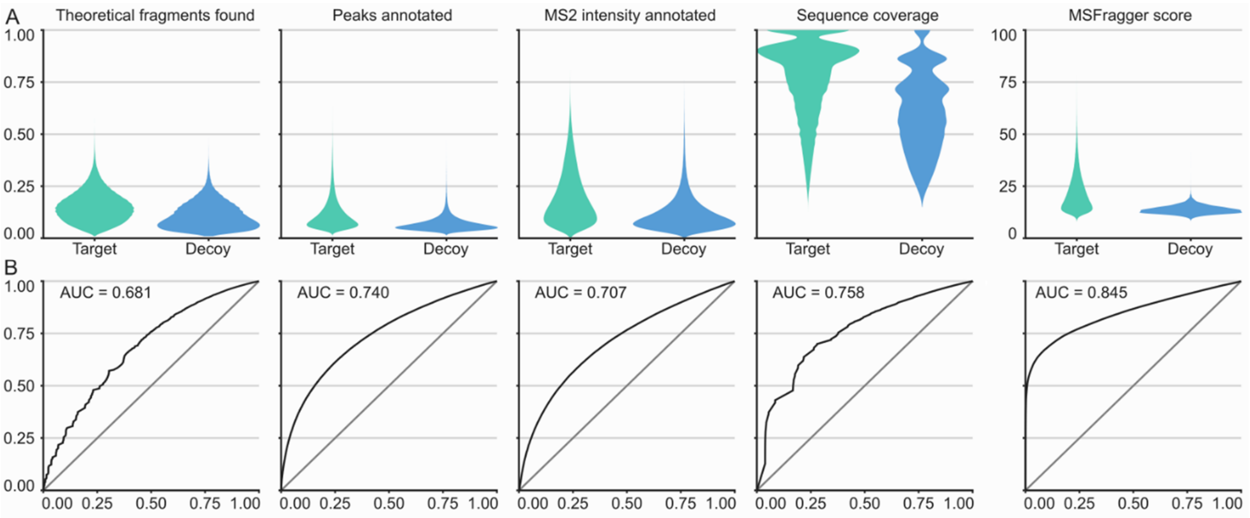

Figure S2: Comparison of annotation statistics with MSFragger. A) Violin plots for each of the annotation statistics. Statistics include the fraction of theoretical fragments found, fraction of measured peaks annotated, fraction of MS2 intensity accounted for by annotated peaks, fraction of sequence coverage, and the MSFragger score. B) For each statistic in A the ROC curve with the area under the curve (AUC) annotated.

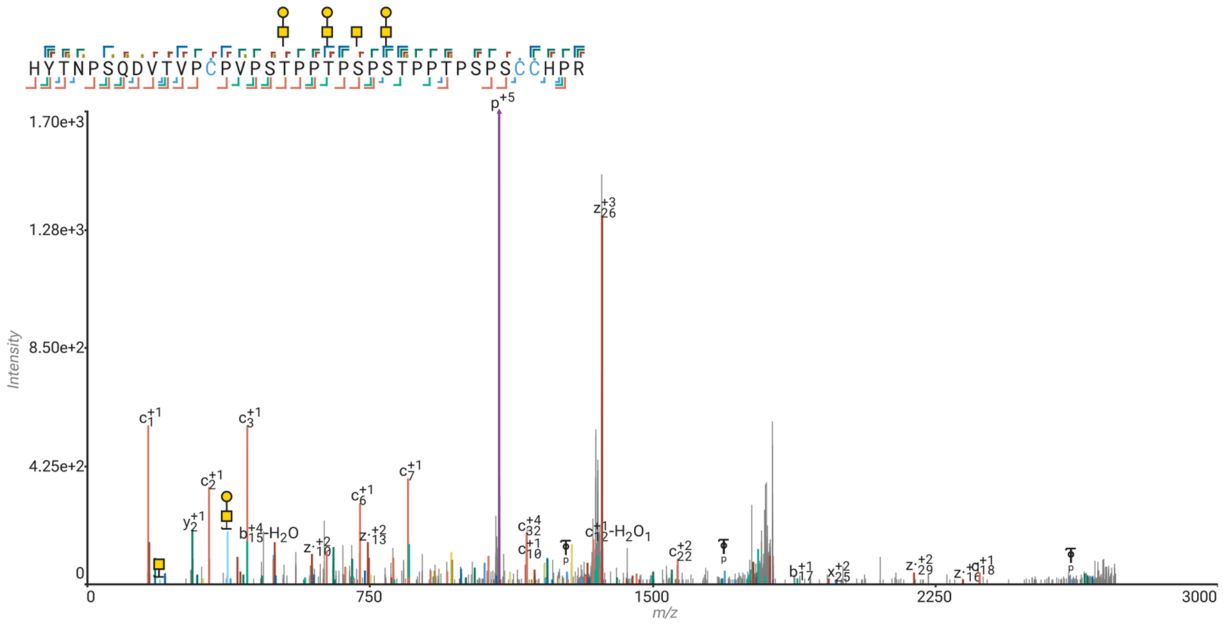

Figure S3: Site-localization of post-translational modifications. Shown is an EACID MS2 spectrum acquired on the ZenoTOF 7600 for a tryptic O-glycosylated IgA peptide, corresponding to the hinge region of the heavy chain. The combined O-glycans total to a glycan composition of HexNAc(4)Hex(3). Localization of the O-glycans was performed manually, aided by Annotator. Each localized glycan is fully supported by an extensive array of c- and/or z-ions. Annotation is performed following the standard nomenclature for B-, Y- and oxonium ions.

Supplementary reference:

(1) Tran, N. H.; Qiao, R.; Xin, L.; Chen, X.; Liu, C.; Zhang, X.; Shan, B.; Ghodsi, A.; Li, M. Deep Learning Enables de Novo Peptide Sequencing from Data-Independent-Acquisition Mass Spectrometry. *Nat Methods* **2019**, *16* (1), 63–66. https://doi.org/10.1038/s41592-018-0260-3.
